# Rapid Development and Rupture of an Infected Intracranial Aneurysm Caused by Streptococcus salivarius Meningitis: A Case Report

**DOI:** 10.7759/cureus.74059

**Published:** 2024-11-20

**Authors:** Natsumi Hata, Takateru Ihara

**Affiliations:** 1 Department of Emergency Medicine, Hyogo Prefectural Amagasaki General Medical Center, Amagasaki, JPN; 2 Department of Pediatrics, Hyogo Prefectural Amagasaki General Medical Center, Amagasaki, JPN

**Keywords:** adult bacterial meningitis, intracranial aneurysm/diagnosis, intracranial infectious aneurysm, spontaneous pneumocephalus, subarachnoid hemorrhage

## Abstract

The clinical manifestation of intracranial mycotic aneurysms (ICMAs), which are rare but carry high risks of rupture and early mortality, remains poorly understood. We present a case of an ICMA that rapidly developed and ruptured after the diagnosis of meningitis caused by *Streptococcus salivarius *(*S. salivarius*), which rarely causes bacterial meningitis. A 54-year-old man presented with a headache that worsened on coughing, without altered consciousness or vomiting. He was diagnosed with pneumocephalus due to a meningoencephalocele, which was detected on contrast-enhanced magnetic resonance imaging; no arterial abnormalities were identified. The day after admission, his headache worsened and the level of consciousness decreased. The cerebrospinal fluid analysis confirmed *S. salivarius *meningitis. On day 2, the patient suddenly became comatose. Head computed tomography revealed subarachnoid hemorrhage; the patient subsequently died. The autopsy revealed a ruptured 10 mm aneurysm in the right vertebral artery. ICMA is a rare but potentially fatal complication of bacterial meningitis. This case highlights the rapid progression of aneurysm formation during treatment, despite improvement of clinical symptoms and normal findings on initial imaging.

## Introduction

Mycotic aneurysms (MAs) develop when infection destroys the arterial wall structure [1]. Infected intracranial aneurysms, known as intracranial MAs (ICMAs), are rarer than other MAs but can be fatal because their rupture might cause intracranial hemorrhage [2]. ICMAs account for 0.5-6.5% of all cerebral aneurysms; the associated mortality rate is 24% [3]. Infective endocarditis (IE) is the most common cause of ICMAs, followed by dental infection and meningitis [4]. The aneurysm formation mechanism due to IE is as follows: An embolus occludes a microvessel, bacteria spill into the arterial inner wall, and inflammation spreads [5]. In contrast, meningitis is a direct extension from a contiguous focus to infecting arterial walls and brain abscesses [6]. ICMAs rupture regardless of size and are often asymptomatic until rupture. The history of and risk factors for ICMAs are unknown because most of the available literature comprises small case series or case reports. We report a rare case of an ICMA secondary to meningitis accompanied by a pneumocephalus and cerebrospinal fluid (CSF) leak. In this case, the aneurysm developed rapidly despite treatment for meningitis caused by *Streptococcus salivarius* (*S. salivarius*), a known commensal bacterium of the oral cavity and gut that rarely causes bacteremia.

## Case presentation

A 54-year-old man presented to the emergency department with a headache and clear nasal discharge that persisted for three weeks. His headache worsened over three days and was triggered by a cough. He had a history of metformin-treated diabetes, immunoglobulin A nephropathy, and asthma, with no history of head or ear, nose, and throat trauma or surgery. His vital signs were axillary temperature, 35.5℃; heart rate, 85 beats/min; blood pressure, 130/92 mmHg; respiratory rate, 16 breaths/min; and oxygen saturation, 95% on ambient air.

**Figure 1:**
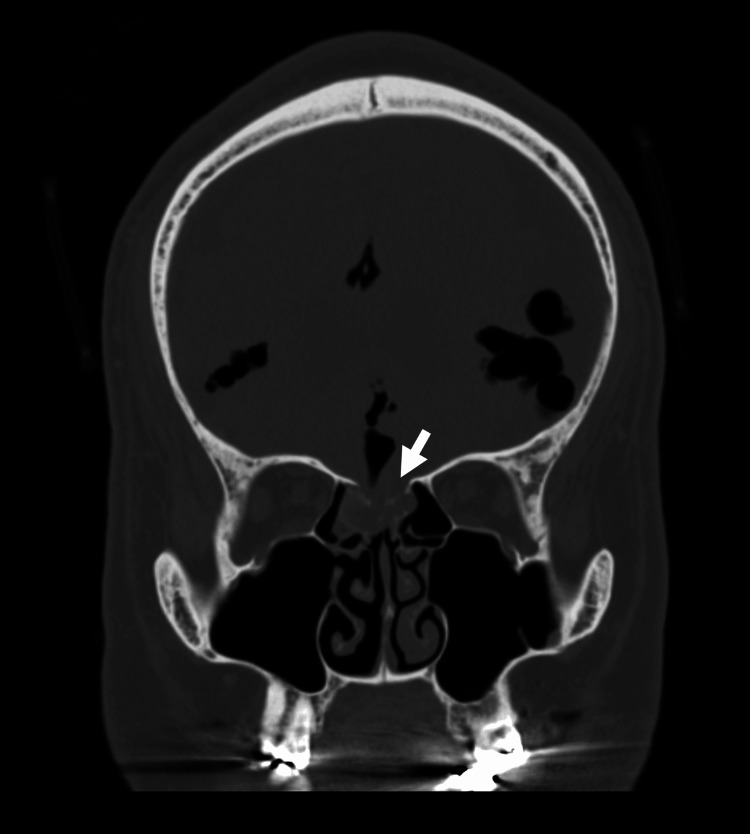
Computed tomography (CT) scan of the head upon admission, revealing a bony defect in the frontal cranial fossa with communication to the ethmoid sinus. The white arrow indicates a bone defect in the anterior cranial fossa.

His Glasgow Coma Scale (GCS) score was E4V5M6, with no neck stiffness, and both Brudzinski and Kernig signs were negative. Non-contrast brain computed tomography (CT) revealed air in the subarachnoid spaces and a bone defect in the anterior cranial fossa communicating with the ethmoid sinus (Figure 1).

**Figure 2:**
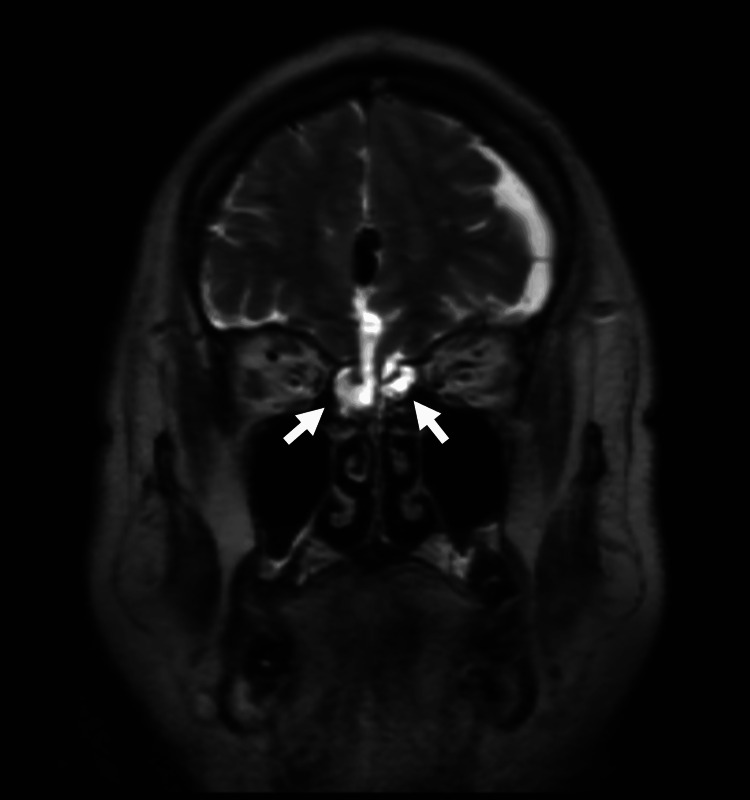
Magnetic resonance imaging (MRI) on admission, demonstrating prolapse of brain parenchymal tissue from the anterior skull base into the ethmoid sinus. The white arrows indicate bone defects at the bilateral skull base and herniation of brain tissue.

Contrast-enhanced magnetic resonance imaging (MRI) revealed brain tissue prolapse into the right ethmoid sinus and a bone defect in the left middle cranial fossa (Figure 2).

No cerebral aneurysm was observed. Laboratory tests showed a leukocyte count of 15.8 × 10⁹/L, creatinine level of 1.29 mg/dL, and C-reactive protein level of 3.47 mg/dL; blood cultures were negative. A dipstick test of the nasal liquid was positive for glucose. Based on these findings, the patient was diagnosed with pneumocephalus due to meningoencephalocele and was hospitalized.

On the second day, his headache worsened and the GCS score decreased (E3V4M5). MRI showed increased signal intensity in the subarachnoid space. A lumbar puncture was performed immediately, and a spinal drainage tube was placed. CSF analysis revealed a yellow, turbid appearance with an elevated leukocyte count (6856/μL, 95% polymorphonuclear cells) and protein level (591 mg/dL), while glucose was undetectable. The red blood cell count was 1/μL. No evidence of subarachnoid hemorrhage (SAH) was observed at this point. Empirical ceftriaxone and vancomycin treatment was administered because bacterial meningitis was suspected. The day after treatment began, his GCS score improved to E4V5M6.

**Figure 3:**
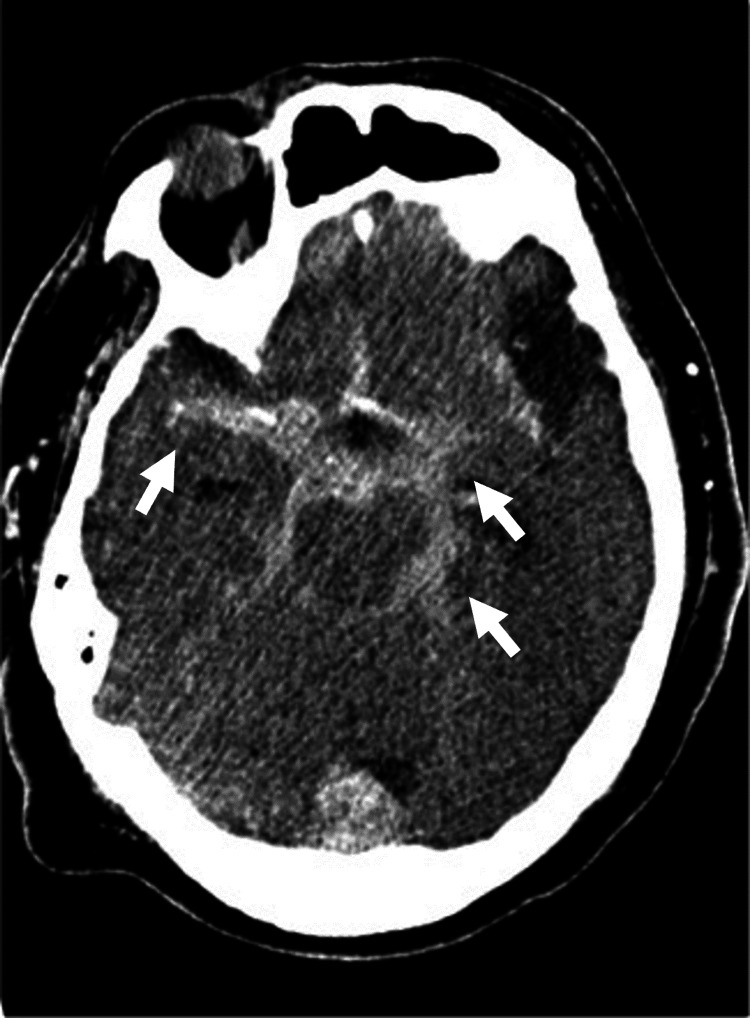
Computed tomography (CT) scan of the head performed when the patient's condition suddenly changed. The white arrows indicate hemorrhage extending into the basal cistern, ambient cistern, and Sylvian fissure, consistent with a diagnosis of acute subarachnoid hemorrhage (SAH).

**Figure 4:**
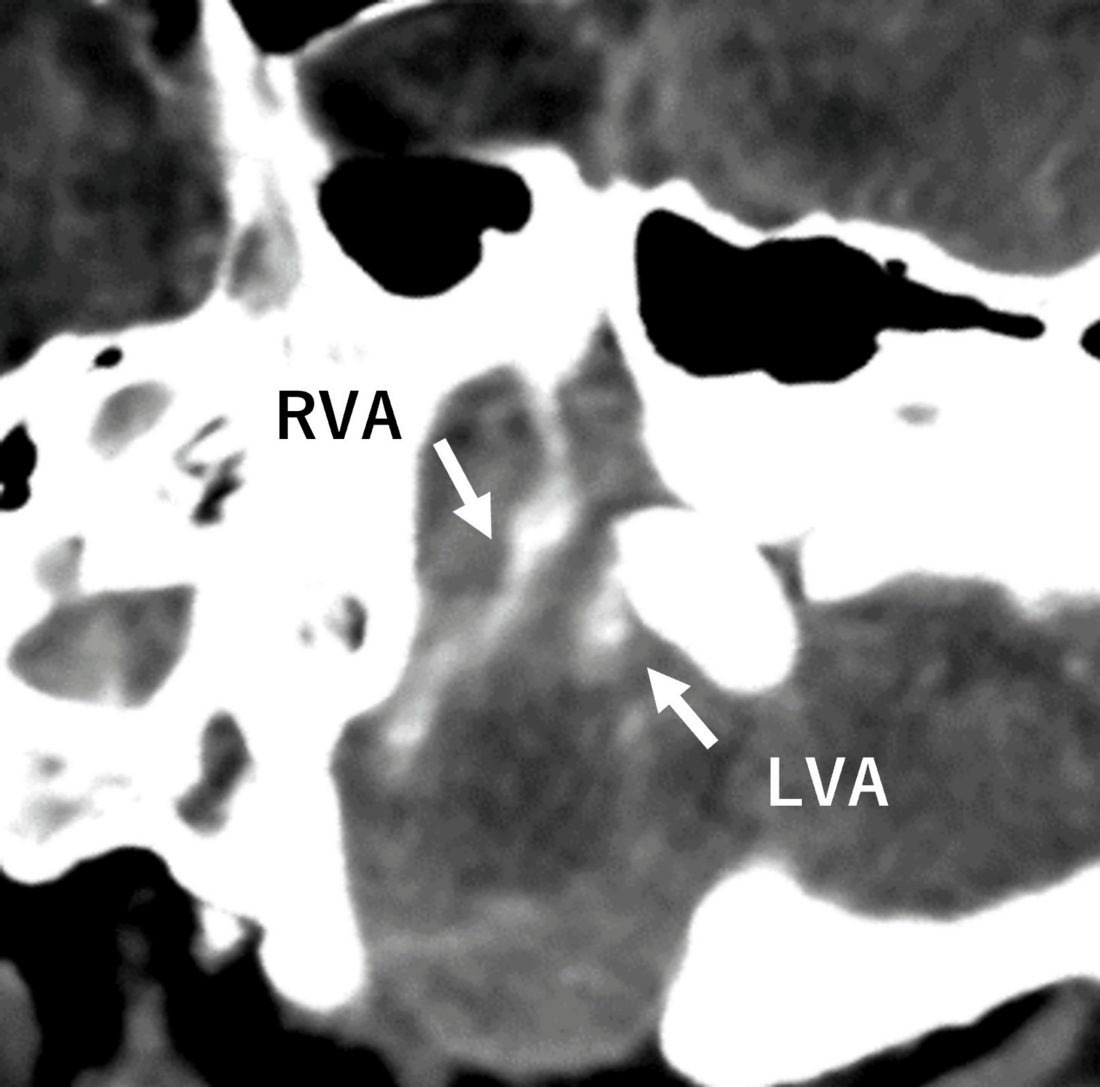
Computed tomography (CT) angiography performed following the non-contrast head CT reveals a ruptured aneurysm in the right vertebral artery, identified as the source of the subarachnoid hemorrhage. LVA: left vertebral artery; RVA: right vertebral artery

On treatment day 2, the spinal tube drainage became hematogenous, and the patient’s GCS score suddenly worsened to E1V1M1: he was in respiratory arrest and his pupils were dilated and non-reactive. At this time, the World Federation of Neurosurgical Societies grading was assessed as Grade V. The head CT showed an SAH (Figure 3), and CT angiography (CTA) revealed a right vertebral artery aneurysm (Figure 4).

**Figure 5:**
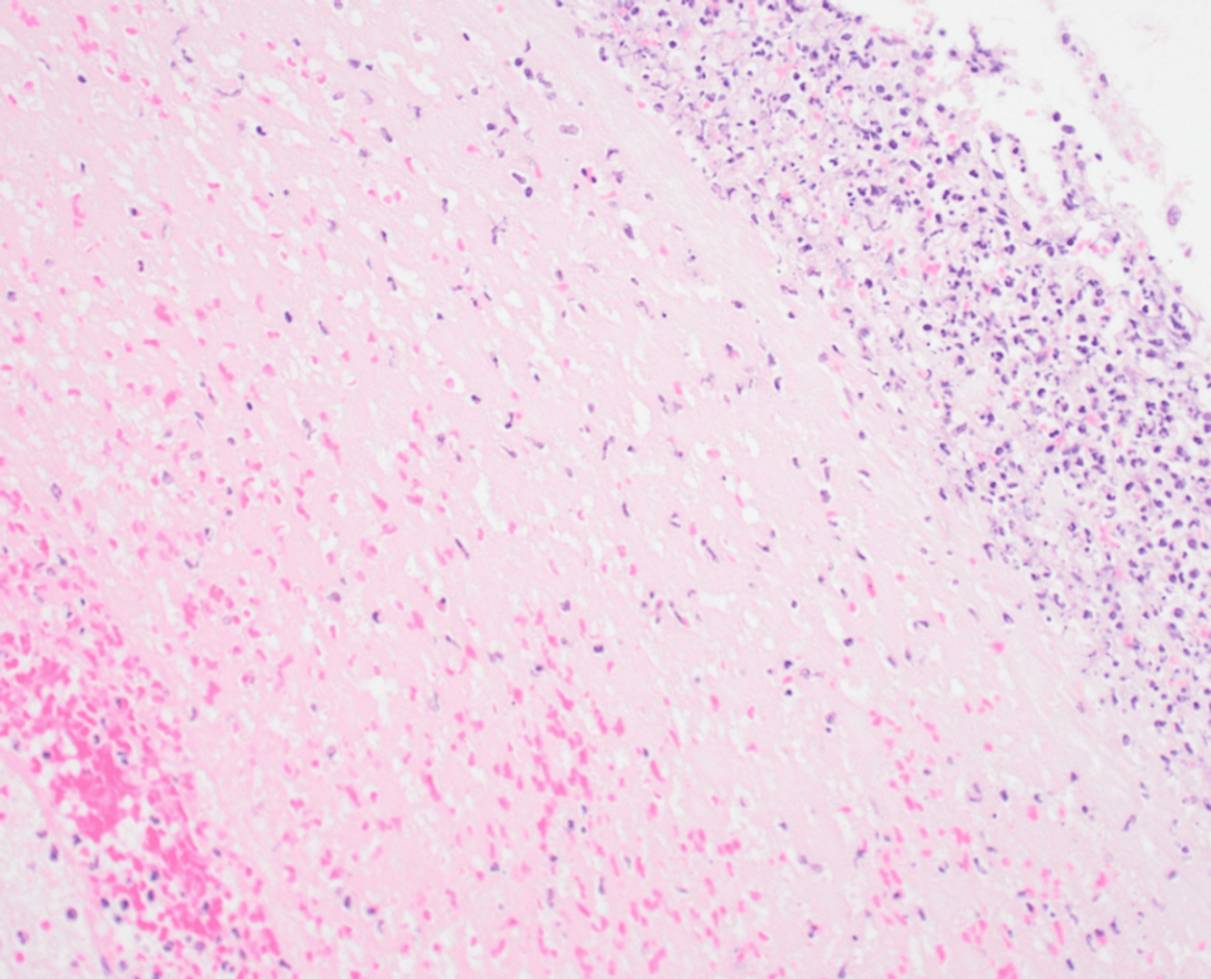
Hematoxylin and eosin staining at 400X magnification reveals the arterial adventitia at the site of the aneurysm rupture, demonstrating significant infiltration of inflammatory cells.

The patient was intubated and transferred to the intensive care unit. Neurosurgical intervention was not expected to alter the outcome because the SAH severity was determined to be Grade 5 according to the Hunt and Kosnik classification. His family decided to withdraw care; the patient died the day after SAH detection. *S. salivarius*, which has good antimicrobial susceptibility, was isolated from the CSF. Identification was performed using the MALDI Biotyper system (Bruker Corporation, Billerica, USA), with a confidence score of 2.32 in a subsequent analysis. The autopsy revealed a 10 mm right vertebral artery aneurysm with rupture and intense inflammatory infiltration around the aneurysm (Figure 5).

## Discussion

This case report describes the rapid formation of an infectious aneurysm caused by *S. salivarius*, a rare cause of bacterial meningitis. Fever, neck stiffness, and change in mental status are the classic triad of bacterial meningitis; however, all three symptoms are present in only 44% of cases. Nevertheless, 95% of patients with bacterial meningitis exhibit at least two of the following four symptoms: headache, fever, neck stiffness, and change in mental status [7]. In a review of 64 cases of *S. salivarius* meningitis by Wilson et al., the clinical symptoms were similar to those of common bacterial meningitis, with 90% of patients presenting to the hospital within one day of symptom onset [8]. Importantly, the aneurysm, which was not observed on initial imaging during the early stages of meningitis, developed rapidly within a short period.

The aneurysm development rate after an infection diagnosis is unclear. However, several case reports have discussed the time from diagnosis to rupture in patients with ICMAs secondary to IE. In 13 ICMA cases, the average time from IE diagnosis to rupture of ICMA was 18 days (range 0-35 days) [9]. Animal studies have shown that infected emboli occlude microvessels, leading to surrounding vasodilation and inflammatory cell infiltration and potentially resulting in aneurysm formation and rupture within 24 hours [10]. They also suggested that infected aneurysms develop slowly when the causative organism is susceptible to antimicrobial therapy. However, there are few reports on the time from infection onset to ICMA rupture resulting from the direct extension of a contiguous focus, such as meningitis. According to these reports, ICMAs can develop rapidly, within one to five days, in cases of bacterial meningitis. Choi et al. reported ICMAs in a patient with meningitis that appeared and ruptured within 24-48 hours, with *S. pneumoniae *detected in the CSF. *S. pneumoniae* is a known cause of invasive central infections with high mortality rates [11]. Meningitis caused by *S. pneumoniae* is associated with a higher rate of cerebrovascular complications than other bacterial meningitis [12]. *S. pneumoniae* invasiveness may be closely related to ICMA development.

In our case, *S. salivarius*, an attenuated bacterium, was detected in the CSF. *S. salivarius* normally inhabits the human oral cavity and is an uncommon pathogen. Most *S. salivarius* infections of the central nervous system are nosocomial or iatrogenic [13]. According to the case series by Wilson et al., the associated fatality rate is 3%, which is lower than that in other bacterial meningitis cases [8]. In our patient, a bone defect in the skull base due to a meningoencephalocele may have caused *S. salivarius* meningitis. ICMAs caused by IE due to *S. salivarius* have been reported [14], but meningitis has not been reported. The rapid formation of an aneurysm in this case may have been influenced by delayed treatment, which has been reported as a risk factor for aneurysm formation [10]. The mild clinical symptoms present at the time of admission could also have contributed to the delay in the diagnosis and treatment of meningitis, potentially leading to aneurysm development.

In this case, it was necessary to consider whether the aneurysm was not detected on initial imaging. In recent years, CTA, MR angiography, contrast-enhanced MRI, and selective catheter digital subtraction angiography (DSA) have been widely used to diagnose aneurysms. DSA is the most reliable imaging technique; however, it is time-consuming and invasive. The sensitivity and specificity of CTA for aneurysms are 97.2% and 97.9%, respectively [15]. In our case, a contrast-enhanced MRI was performed upon admission. The sensitivity of contrast-enhanced MRI compared with DSA was 100% when the aneurysm was ≥3 mm in size [16]. Therefore, the possibility of overlooking an aneurysm is extremely low because a 10 mm vertebral artery aneurysm was identified on CTA and autopsy.

## Conclusions

ICMA is a rare but potentially fatal complication of bacterial meningitis. This case emphasizes the rapid progression of aneurysm formation during the treatment of bacterial meningitis associated with pneumocephalus. While early detection of aneurysms is crucial, the risk of aneurysm formation cannot be overlooked even when clinical symptoms improve and no abnormalities are detected on initial imaging. Careful monitoring of symptoms may aid in early detection, but timely diagnosis and interventions to improve prognosis remain challenging.
